# The p53HMM algorithm: using profile hidden markov models to detect p53-responsive genes

**DOI:** 10.1186/1471-2105-10-111

**Published:** 2009-04-20

**Authors:** Todd Riley, Xin Yu, Eduardo Sontag, Arnold Levine

**Affiliations:** 1 The Institute for Advanced Study, Princeton, New Jersey, USA; 2 The BioMaPS Institute at Rutgers University, Piscataway, New Jersey, USA; 3 The Mathematics Department, Rutgers University, Piscataway, New Jersey, USA; 4 The Cancer Institute of New Jersey, New Brunswick, New Jersey, USA

## Abstract

**Background:**

A computational method (called p53HMM) is presented that utilizes Profile Hidden Markov Models (PHMMs) to estimate the relative binding affinities of putative p53 response elements (REs), both p53 single-sites and cluster-sites. These models incorporate a novel "Corresponded Baum-Welch" training algorithm that provides increased predictive power by exploiting the redundancy of information found in the repeated, palindromic p53-binding motif. The predictive accuracy of these new models are compared against other predictive models, including position specific score matrices (PSSMs, or weight matrices). We also present a new dynamic acceptance threshold, dependent upon a putative binding site's distance from the Transcription Start Site (TSS) and its estimated binding affinity. This new criteria for classifying putative p53-binding sites increases predictive accuracy by reducing the false positive rate.

**Results:**

Training a Profile Hidden Markov Model with corresponding positions matching a combined-palindromic p53-binding motif creates the best p53-RE predictive model. The p53HMM algorithm is available on-line:

**Conclusion:**

Using Profile Hidden Markov Models with training methods that exploit the redundant information of the homotetramer p53 binding site provides better predictive models than weight matrices (PSSMs). These methods may also boost performance when applied to other transcription factor binding sites.

## Background

**Figure 1:**
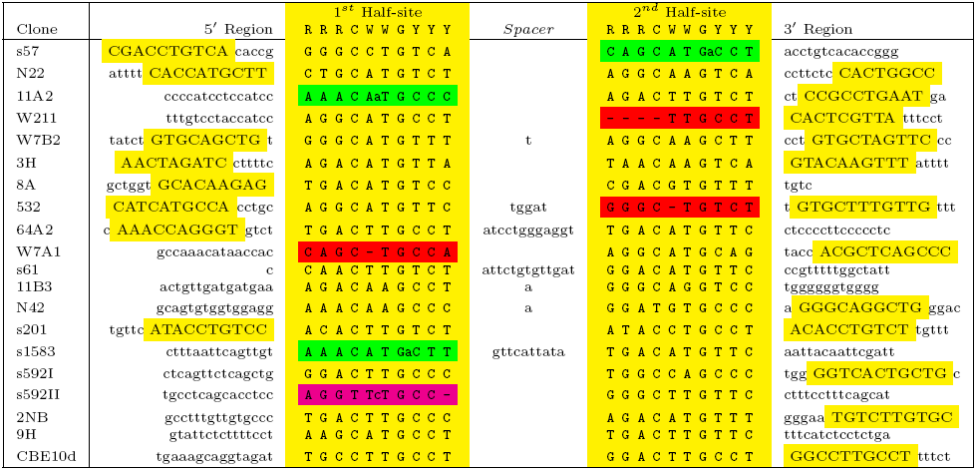
**Original Data from El-Deiry et al., Used To Define The p53 Consensus Binding Site**. The original DNA fragments collected from a genome-wide, p53-antibody immunoprecipitation, that were used to define the head-to-head (HH) p53 Consensus Binding Site, are graphically presented [3]. The yellow columns corresponding to the 1^*st *^and 2^*nd *^half-sites were used to define the consensus p53 motif. The p53 binding site is highly degenerative. Within the yellow columns, notice that 7 of the 20 DNA target sites (35%) had at least one nucleotide insertion (green), deletion (red), or both (magenta) relative to the discovered 10 bp-spacer-10 bp consensus. Since insertions and deletions throw off the reading frame of a weight matrix, any PSSM approach will inherently mis-score at least 35% of these 20 sites. Alignments of the 160 experimentally validated p53 binding sites also reveal that any PSSM approach would inherently mis-score at least 30% of them as well. Another observation is that additional p53 half-sites are immediately adjacent (in yellow) to the ones used to define the consensus in 15 of the 20 target sites (75%). Since the genome-wide immunoprecipitation study was designed to pull down the highest affinity sites, the fact that 75% of the target sites are actually p53 cluster-sites is the first indication that cluster-sites of 3 or more half-sites confer higher binding affinity [22].

The p53 protein plays a crucial role in cancer suppression in the human body. In response to cancer-inducing, DNA-damaging stress conditions, the tetrameric p53 proteins can activate different pathways that lead to DNA repair, cell cycle arrest, inhibition of angiogenesis, and apoptosis [1]. A highly degenerative, palindromic consensus DNA binding site, consisting of a half-site RRRCWWGYYY, followed by a variable length spacer, then followed (almost always) by a second half-site RRRCWWGYYY sequence, has been discovered for the protein, where R is a purine, Y a pyrimidine, W is either A or T (adenine or thymine) and G is guanine and C is cytosine (see Figure 1) [2,3]. By labeling each quarter-site RRRCW as → and its reverse-complement WGYYY as ←, the first discovered p53 consensus sequence can be graphically represented by → ← *spacer *→ ←. This configuration of the four quarter-sites is often referred to as the head-to-head (HH) orientation, and represents the vast majority of experimentally-validated p53 binding sites to date.

### The degeneracy of the p53-RE

In the influential paper "Definition of a Consensus binding Site for p53", by El-Deiry et al., 7 of the 20 DNA target sites (35%) used to form the head-to-head (HH) p53 consensus sequence had at least one nucleotide insertion or deletion relative to the discovered 20 bp consensus after proper alignment (see Figure 1) [3]. Alignments of the roughly 160 experimentally-validated p53 binding sites to date also show that approximately 30% of presently known sites have at least one nucleotide insertion or deletion relative to the consensus matrix [4]. Discovery of p53 binding sites with such degeneracy cannot be reliably made with a PSSM approach, since prevalent insertions and deletions in the consensus sequence misalign the PSSM reading frame, and lead to improper scoring. Therefore, PSSM binding site discovery algorithms inherently mis-score at least 30% of the known p53 binding sites.

### PHMMs can model nucleotide insertions and deletions

**Figure 2:**
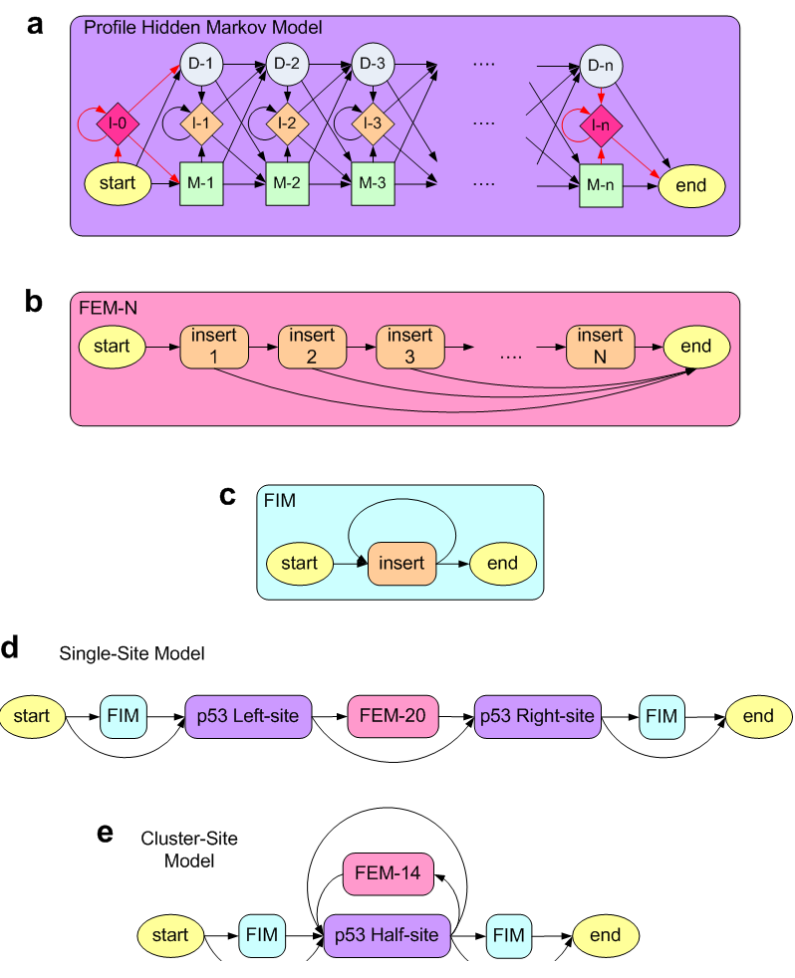
**The Topologies of p53 Single-site and Cluster-site Models**. **(a) **A Profile Hidden Markov Model (PHMM) contains three hidden states for each position in a sequence motif of length *n*: a match state (green squares), an insertion state (orange diamonds), and a delete state (gray circles). The arrows represent allowed transitions between states and have associated probabilities. The match and insertion states also have associated nucleotide emission probabilities. The first and last insertion states (I-0 and I-n) and associated transitions (in red) are shown for completeness. However, they are not present in the p53 models since they are replaced by FIM and FEM models. **(b) **The topology of the Finite Emission Module (FEM) of length *N *allows the ability to model any distribution of spacer-lengths between 1 and N. For the p53 models, the model and background probabilities within the FEM modules are identically uniform so that there is no-cost for spacer-lengths between 1 and *N*, and are referred to as "no-cost FEMs". **(c) **The topology of the Free Insertion Module (FIM) allows for the ability to model an exponentially decaying distribution of spacer-lengths. However, by setting the model and background probabilities to identically uniform, the FIM can model any sequence of infinite length with no associated cost to the overall score (hence the word "Free"). **(d) **The main components of the p53 single-site model are the left and right half-site PHMMs, which potentially contain corresponding positions between them. These two half-site models are separated by a no-cost FEM model that limits the length of any intervening spacer sequence to 20 bp. The half-site models are also wrapped by two FIMs that allow the Viterbi algorithm to find the best matching motifs anywhere in the candidate sequences. **(e) **The topology of the p53 cluster-site model consists of a single PHMM that models a general half-site, and two back-transitions that allow for modeling an infinite number of half-sites within the cluster-site. The back-transition through the no-cost FEM-14 model limits the spacer-sequence between the half-sites to lengths ≤ 14 bp.

Profile Hidden Markov Models provide a coherent theory for probabilistic modeling of degenerate binding sites where random nucleotide insertions into and deletions from the motif are tolerated at certain positions [5,6]. Natural selection suggests that critical nucleotides are conserved over evolutionary time, while non-critical nucleotides (including tolerated insertions in the motif) are not conserved. The match-state emissions of the PHMM serve to model the critical positions in the motif with their observed nucleotide frequencies. The additional hidden deletion and insertion states at each position enable the model to train for (relatively rare) observed deletions and insertions at different positions in the motif (see Figure 2). Although the probability of any particular insertion or deletion of a nucleotide at a certain position in a functional motif may be rare, the accumulated probability over all the positions in the motif that an insertion or deletion event may occur can be significant. The training set of observed insertions and deletions serves to fine-tune the model to be properly sensitive to tolerated deviations from the most prevalent consensus motif. The main strength of the PHMM is this *trained flexibility *to properly model variable length motifs. The major drawback is that more data is required to train the extra parameters not found in weight matrices (PSSMs).

### Using PHMMs to estimate binding affinities

Like weight matrices (PSSMs), Profile Hidden Markov Models can be used to estimate the relative binding affinity of a protein for a particular binding site sequence [7]. Under ideal conditions, the log-odds scores *G*^*s*^(*x*) that a Profile Hidden Markov Model (trained on training set *S*) calculates for any candidate site *x *is directly proportional to the free energy -Δ*G*(*x*) of the TF-protein binding to that candidate site [see Additional file 1 for details] [7-9]. The log-odds scores are given by:



where we define:

(1)

With these definitions, and assuming independence of positions, we have:



The dynamic programming *forward *and *backward *algorithms are used to calculate the probabilities *P*_*hmm*_(*x*) and *P*_*hmm*_(*j, b*). These two probabilities are calculated by summing up the probability of observing the sequence *x*, and the base *b *at position *j*, for all the paths through the linear PHMM, respectively. The dynamic programming *Viterbi *algorithm is used to find the best alignment of the candidate site *x *to the binding-site motif modeled by the PHMM. The best (optimal) alignment of the sequence *x *is obtained by finding the path through the PHMM that gives the highest log-odds score for the sequence [8]. In the case of transcription factor binding sites, the log-odds score of this optimal path (also called the *Viterbi score*) is commonly used to provide adequate approximations to the probabilities *P*_*hmm*_(*x*) and *P*_*hmm*_(*j, b*) [see Additional file 1 for details]. When using the *Viterbi score *for the probability *P*_*hmm*_(*x*) we are assuming that there is generally only one major set of binding interactions between specific nucleotides and amino acids for a given protein-DNA complex, and that all other possible binding locations in the response element can be ignored.

### Training a PHMM with validated binding sites

Before a PHMM can be used to estimate the relative binding affinity for any putative binding site, the PHMM must be trained to properly model a functional binding site of interest. When training a PHMM for a particular motif, the goal is to choose the parameters of the model in order to maximize the likelihood of the sequences in the training set, without over-fitting. Again, under ideal conditions the log-odds score (*log-likelihood ratio*) *G*^*s*^(*x*) to be maximized for the collection of binding sites in the training set is proportional to the estimated binding free energy -Δ*G*(*x*) of these binding sites. When the state paths for the training sequences are not known, no known closed form solution exists for the parameter estimations [8]. The *Baum-Welch *algorithm is the most commonly used iterative Expectation Maximization (EM) method to train the parameters of the model. The Baum-Welch algorithm always climbs the gradient (to increase the combined scores of the training set) and uses the optimized dynamic programming *forward *and *backward *algorithms [8].

## Results and discussion

### A novel training method that boosts predictive power

**Figure 3:**
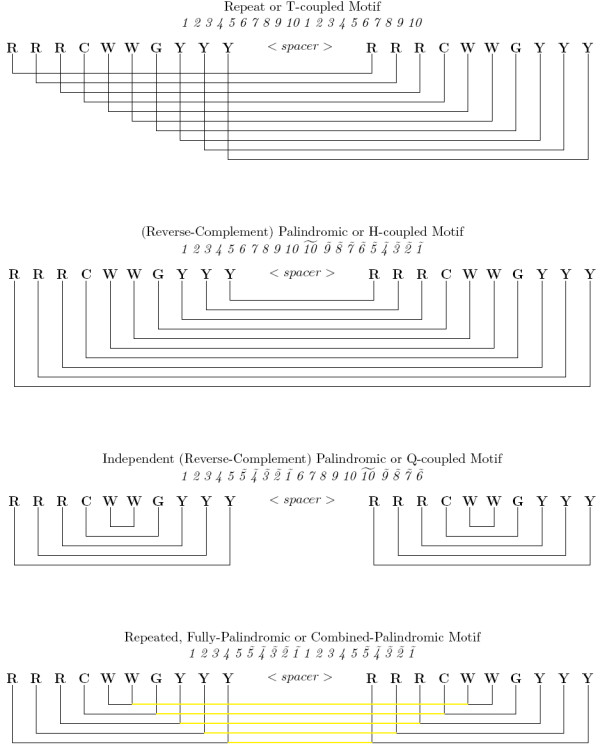
**The Four p53 Correspondence Motifs**. The four correspondence motifs for the repeated, palindromic p53 RE are graphically represented. In the top three motifs, each line corresponds 2 synonymous positions. In the bottom motif, the previously independent half-sites are made corresponding (tied) by the yellow connecting lines so that now 4 synonymous positions are corresponded. The completely un-tied motif (not shown) has no correspondence, and thus no connecting lines, between any of the positions in the motif. (R = A or G, W = A or T, and Y = C or T. Position *ã *has the complement nucleotide emission distribution of *a*.)

**Figure 4:**
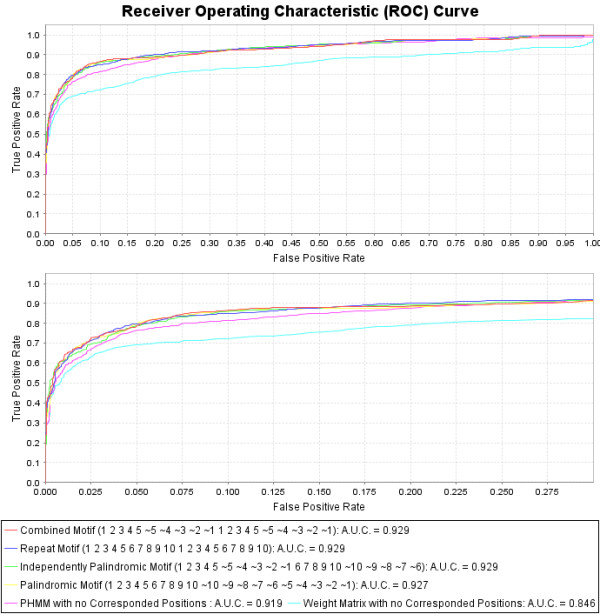
**Cross Validation with Receiver Operating Characteristic (ROC) curves reveals increased predictive power over weight matrices**. 1000 iterations of 10-fold random-split cross validation reveal that the most predictive models utilize the correspondence structures. The combined-palindromic model is the best model since it contains roughly half as many parameters as the other three correspondence models. The positive set contains 160 experimentally validated p53 binding sites, and the negative set contains 40 bp random samples from the mononucleotide content of the training set. The true positive and false positive rates are calculated and plotted for all possible threshold values for each model. The predictive measure for comparing the curves is the AUC (Area Under the Curve). In all the PHMM models the insert-state emissions are fixed to the A, G, C, T nucleotide distribution of the training set. The best classifier uses the combined-palindromic training motif. (Position *ã *has the complement nucleotide emission distribution of *a*).

To increase the predictive power of our p53-motif PHMMs, we attempt to exploit the *a priori *knowledge that when proteins bind as homodimers or homotetramers, their corresponding binding sites typically have a *palindromic*, *repeat*, and/or *reverse complement *structure (see Figure 3). This prior knowledge can be used to correspond (fully or partially tie) the parameters between positions in order to exploit the inherent redundancy in the information of the motif. Within a set of corresponding positions, the updating of emission and transition probabilities can borrow strength from each other by sharing information. In addition, the degree of sharing of information for any set of corresponding positions can be optimized during training. The process of corresponding parameters can greatly reduce the parameter search-space during the training of the model, and provide the ability to train for rare occurrence insertion and deletion events. This general technique has been effectively used when HMMs have been applied to speech and handwriting recognition problems, and has been referred to as *parameter tying *[10]. We introduce an extension to this method that allows for the setting or training for an optimal level of partial or full parameter tying. In the domain of protein-DNA binding sites, even if a palindromic, repeat, or reverse complement structure of a binding site is not known *a priori*, all the known structural motifs can be tested, and the structure can be *discovered *(inferred) from the ROC curve that maximizes predictive accuracy. For example, of the six structural models tested for the p53 binding motif, the combined-palindromic motif that completely corresponds the four quarter-sites is the *discovered *motif, since it is the best classifier (see Figure 4).

### The Corresponded Baum-Welch algorithm

In order to include the prior knowledge of the structural motif (or in an attempt to discover it), a novel "Corresponded Baum-Welch" algorithm is proposed to enforce or learn the optimal correspondence between expectations of parameters for corresponding positions after each iteration of the Baum-Welch algorithm (see Methods). For example, assume that we have prior knowledge that a transcription factor protein binds to the DNA in homodimer form, where each monomer interacts with 5 DNA base pairs. Then a corresponding palindromic motif for the nucleotide positions would be: *1 2 3 4 5 5 4 3 2 1*, while a reverse-complement palindromic motif would be: *1 2 3 4 5 * (where *ã *has the complement nucleotide emission distribution of *a*). All the emission distributions for each of the five sets of synonymous positions would be made corresponding, as well as all the transition probabilities between synonymous positions. In this example, if all the parameters between synonymous positions were fully corresponding (tied), then the parameter search space would be roughly cut in half. The level of correspondence between the parameters for synonymous positions can be given *a priori*, or trained for if the training set is sufficiently large. One optimal level of correspondence, *c*, can be calculated for the whole motif (for all the corresponding positions), or a separate one can be found for each set of corresponding positions. (See Methods for details.)

### Comparing the different p53 corresponding (structural) motifs

Since the 20 bp-tetrameric p53 binding site has a repeated and nested palindromic structure, different correspondence motifs can be constructed to train the PHMM models, and cross validation can be used to compare their predictive properties. The motifs that are compared are: the repeat or T-coupled motif (*1 2 3 4 5 6 7 8 9 10 1 2 3 4 5 6 7 8 9 10*), the (reverse-complement) palindromic or H-coupled motif (*1 2 3 4 5 6 7 8 9 10 *), the independently (reverse-complement) palindromic or Q-coupled motif (*1 2 3 4 5 **6 7 8 9 10 *, the repeated, fully-palindromic or combined-palindromic motif (*1 2 3 4 5 **1 2 3 4 5 *), and the completely un-tied motif with no correspondence between any positions (see Figure 3) [11]. We perform 1000 iterations of ten-fold random-split cross validation on each model to gain statistics on their predictive accuracy. The positive set contains 160 experimentally validated p53 binding sites from [4], and the negative set contains 40 bp random samples from the mononucleotide content of the training set. Then we utilize Receiver Operating Characteristic (ROC) curves in order to compare the predictive power of the classifiers in an unbiased, threshold-independent (non-parametric) manner. This is achieved by calculating the true positive and false positive rates for all possible threshold values for each model. The summary statistic for comparing the ROC curves is the AUC (Area Under the Curve). AUC values lie somewhere between 1.0 and 0.5 (where an AUC of 1.0 would correspond to a perfect classifier, and an AUC of 0.5 would correspond to a classifier that is no better than random coin flipping.)

### Training Insert-State Emissions

A major consideration when training Profile Hidden Markov Models (PHMMs) is which parameters to train for at each position, and which parameters to fix at each position to the over-all average. The more non-fixed parameters that must be trained for at each position in the motif, the more data that is needed to properly train the model. Ideally, a sufficiently large training set is available to be able to train for all the parameters in the PHMM at each position. Unfortunately, in the case of transcription factor binding sites, this is rarely the case. Typically, when using PHMMs to model DNA binding sites, both the insert probabilities and insert state nucleotide emissions probabilities are set to the binding site averages, since there are rarely enough examples of these rare occurrence events at a particular position to train those parameters for that position alone [12]. By corresponding (fully or partially tying) positions and in effect increasing the training data for each position, it may be possible to train the insertion-state emissions distributions for these corresponding positions. This could possibly boost predictive power of the models, if the p53 protein is selective as to which nucleotides can be inserted into the motif at certain positions without compromising the binding affinity of the site. A common example of such selective sequence insertions can be found in functional protein families, whereby hydrophobic or hydrophilic amino acid insertions may be tolerated at certain positions, provided that the insertions are present either in the core or at the surface of the protein, respectively, after folding. Notice that fixing the insertion-state emission distributions at every position to the amino-acid average for the whole sequence would be very inappropriate in this example.

### The final results

**Figure 5:**
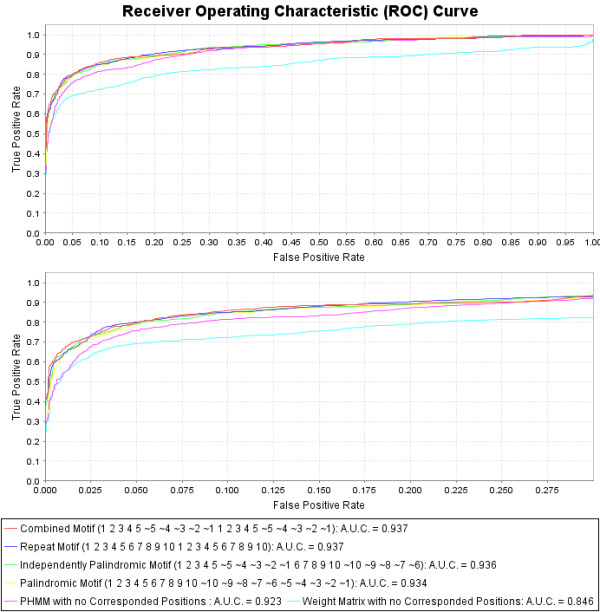
**Cross Validation with Receiver Operating Characteristic (ROC) curves reveals increased predictive power when training insert-state emissions**. All the PHMM models in this comparison train the insert-state emission distributions based on positional insertions occurring in the training set. Again, 1000 iterations of 10-fold random-split cross validation reveal that the most predictive models utilize the correspondence structures. The positive set contains 160 experimentally validated p53 binding sites, and the negative set contains 40 bp random samples from the mononucleotide content of the training set. The true positive and false positive rates are calculated and plotted for all possible threshold values for each model. The predictive measure for comparing the curves is the AUC (Area Under the Curve). The AUC values improve for all the PHMM models compared to Figure 4, but not for the weight-matrix model (which does not use the insert states). The best classifier (with the combined-palindromic training motif) was used for the p53HMM algorithm. (Position *ã *has the complement nucleotide emission distribution of *a*).

The combined-palindromic motif (*1 2 3 4 5 **1 2 3 4 5 *) performs on par with or better than all other structural motifs, although it contains comparably half the degrees of freedom (see Figures 4 and 5). In addition, all four of the structural motifs perform on par with each other. These results suggest that there exist correlations between the positions in the repeat, independently palindromic, and palindromic motifs, and that the combined-palindromic motif leverages the correlations found in all of them. Furthermore, it can be seen that training the insert-state emissions per corresponding position also boosts the predictive power of all the models (see Figures 4 and 5). Analysis of the AUC measurements reveals some interesting features. Adding insert-state emission training to the base PHMM (with no motif-corresponded positions) has an AUC improvement of .923 - .919 = .004, but with motif training has one of .937 - .929 = .008. Adding motif training (motif-corresponded positions) to the PHMM when not insert-state emission training has an AUC improvement of .929 - .919 = .010, but with insert-state emission training has one of .937 - .923 = .014. Therefore the improvements are not additive. There is "positive synergy" when performing both motif training and insert-state emission training together that further boosts the predictive accuracy of the model. This observation confirms our hypothesis that training insert-state emissions can significantly boost the accuracy of the model after corresponding positions in the PHMM according to a binding-site motif.

**Figure 6:**
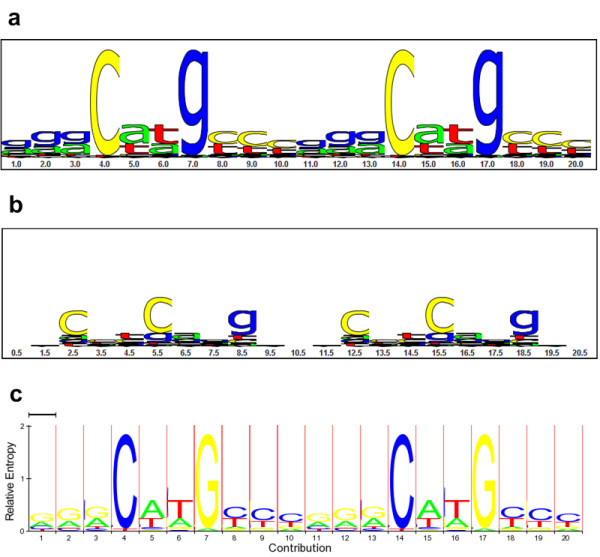
**The p53HMM Match and Insert Emissions**. **(a) **The match-state sequence logo for the combined-palindromic p53 motif: *1 2 3 4 5 **1 2 3 4 5 *. (Motif position *ã *has the complement nucleotide-emission distribution of *a*.) The height of each letter is made proportional to its frequency at each position, and the letters are sorted in descending frequency order. The height of the entire stack at each position is then adjusted to signify the information content (in bits) of that position [25]. The match-state nucleotide positions 4, 7, 14, and 17 (motif positions *4*, *7*, , and  respectively) are the most conserved and are the main points of contact with the p53 protein. **(b) **The insert-state sequence logo for the same combined-palindromic p53-model. These nucleotide insertions occur in-between the nucleotide positions shown in part **a**. The specificity motif of the insert-state emissions is different from that of the match-state emissions. **(c) **The HMM logo that combines parts **a **and **b **and state transition information into one graph. The wide, white-background stacks correspond to the match states in part **a**, while the narrow, red-background stacks correspond to the insert states in part **b**. (A weakness of this HMM logo is that the insert-state stacks are so narrow that it is difficult to accurately see the stack specificity depicted in part **b**.) The y-axis is the same for all three graphs. However, the width of a stack in the HMM Logo is proportional to the expected contribution of that match or insert state to an emitted sequence of the model [26].

In addition, the more correspondence placed between the synonymous positions during each training iteration, the better the resulting classifier at that point in the training (results not shown). For this training set, all the combined-palindromic models with fixed correspondence factors between *c *= 0.4 and *c *= 1.0 eventually converged to the same predictive model, although lower correspondence factors required more iterations to do so. All the models converged on correspondence factors between *c *= .98 and *c *= .999 when training for optimum correspondence. Therefore the best predictive model completely corresponds (ties) the four quarter-sites in a combined-palindromic structure during each iteration of the training. Our published p53HMM algorithm is this best predictive model: trained on the dataset of 160 functional p53 REs, fully corresponding the data per position based on the combined-palindromic structural motif, and training the insert-state emissions (see Figure 6).

### Validation of the p53HMM algorithm

The new p53HMM algorithm was used to screen for putative p53 binding sites in the endosomal compartment genes, which led to the discovery of a functional p53 site and a new p53-regulated gene, CHMP4C [13]. The putative p53RE sequence AAACAAGCCC agtagcagcagctgctcc GAGCTTGCCC was predicted in the promoter region (-497 to -460 bp) of the CHMP4C gene. The data from the chromatin immunoprecipitation and the luciferase reporter assays showed that p53 protein can bind to this sequence and induce CHMP4C gene expression. Additionally, analysis by p53HMM found an alternative putative p53 binding site in the LIF gene that corresponds to a 6 bp upstream shift of the downstream half-site relative to the recently published putative site in intron 1 [14]. The p53HMM algorithm predicted the site GGACATGTCGGGACA-GCTC, which matches the consensus RRRCWWGYYYRRRCWWGYYY perfectly except for the low-conserved position 10 and the gap ("-", deletion) at position 16. A PSSM approach predicted the shifted site GGACATGTCGggacagCTCCCAGCTC, which is the best "gap-less" p53 site in the region conferring p53 regulation, but it still matches the consensus very poorly with five mismatches (the putative spacer sequence is in lowercase) [14]. A few genes in the dataset of 160 functional p53 binding sites have a deletion relative to the consensus exactly between the well-conserved C and G as seen above, including the genes: EGFR, TYRP1, EEF1A1, HSP90AB1, and BAI1. This discovery of an alternative p53 binding site that better matches known functional sites, by modeling for observed insertions and deletions, highlights some of the advantages of the new p53HMM algorithm.

### Special considerations for the p53HMM algorithm

Although the spacer within a p53 RE has been shown to greatly affect the binding affinity for p53 protein, the ability to properly quantify this effect for all possible spacers of lengths 0–21 base pairs has been elusive. Therefore like previous algorithms, we have chosen to initially ignore the spacers of the training set and putative REs [15]. We are able to ignore arbitrary-length spacers by inserting a no-cost *Free Insertion Module *(FIM) between the two half-sites of the single-site PHMM [16,17]. Similarly, we can ignore spacers with lengths between 1 and *N *base pairs by inserting a no-cost *Finite Emission Module *(FEM-N) between the two half-sites (see Figure 2). A prior p53 RE search algorithm (p53MH) was based upon a PSSM approach and a novel filtering matrix [15]. Unfortunately, the tables were not symmetric and the filtering table over-fit the available data at the time. The combined result was that the p53MH method completely rejects 58 of the 160 experimentally validated sites to date (receiving a score of 0 out of 100, where 100 represented the maximum relative binding affinity). Additionally, some sites received very high scores approaching 100, while the reverse-complement received a score of 0, and vice-versa. Due to these observations, we have purposely designed the p53HMM algorithm to be symmetric, so as to give identical scores for putative sites and their reverse complements. Secondly, we chose to abandon the filtering matrix to avoid over-fitting the available data. A feature that we preserved from p53MH is the normalizing of scores by the highest possible affinity for the motif (×100), so that the highest possible normalized score is 100.

### Modeling dependencies between positions

PSSMs assume that all nucleotide positions within the motif contribute independently to the binding affinity of the binding site, which has been shown experimentally to not always be the case [7]. Recent research has focused on modeling dependencies between positions in protein-DNA binding sites [18,19]. Typically *Tree Bayesian Networks *and *Mixtures *of trees have been used to attempt to model these dependencies between positions, which have been shown through cross validation to increase the predictive power of these models [18]. Our PHMM models do not attempt to model dependencies between the positions, however they can be extended to do so by using higher-order Profile Hidden Markov Models. Unfortunately, the ability to train for positional dependencies, and boost predictive power, is dependent upon the sampling size of the training set and requires larger training sets to train the extra parameters.

### A novel p53 cluster-site algorithm

**Figure 7:**
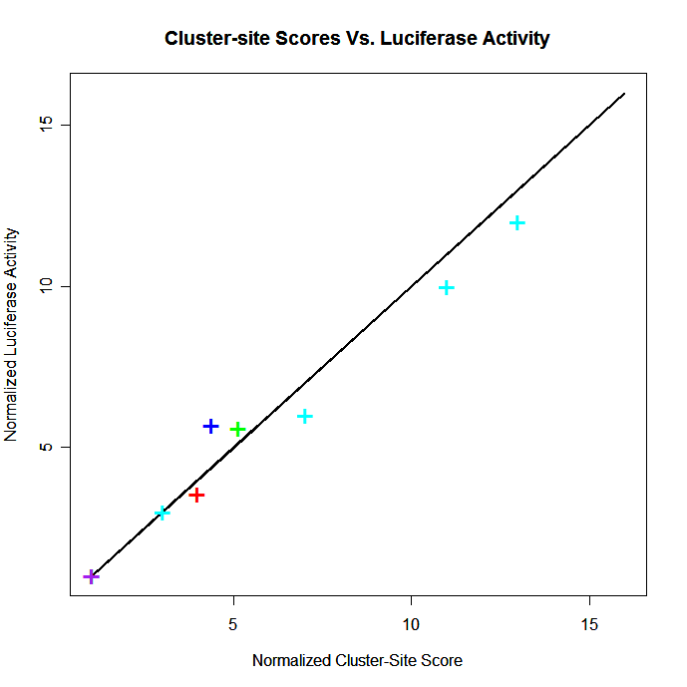
**Comparison of Cluster-site scores and Luciferase Activity**. This graph compares the estimated relative binding affinity given by the cluster-site score to the luciferase activity from four experiments for four different p53 cluster-sites. The four cluster-sites regulate the genes DDB2 (blue), CKM (red), IGFBP3 (green), and TP53I3 (cyan). In all four experiments the luciferase activity of truncated mutants of the respective p53 cluster-site were compared to the luciferase activity of the full cluster-site. In the case of the TP53I3 cluster-site, four different mutants of varying lengths were measured for luciferase activity. All cluster-site scores and activity measurements are normalized by the full-site (two half-sites) measurement. The cluster-site scores are attained by summing the estimated binding affinity of all viable full-sites in the cluster-site that have an affinity above a lower bound and spacer-lengths below an upper bound. The full-site affinity lower bound and spacer-length upper bound were chosen to best match the experimental data. The best fit was attained by enforcing that spacer-lengths not exceed 14 bp and affinity scores exceed 27.5.

Binding affinity measurements have been obtained for certain p53 cluster-sites of different lengths by mutating or truncating known p53 cluster-sites in the genes: DDB2, TP53i3, CKM, IGFBP3, and RGC (see Table 1 and Figure 7) [20-23]. Based on the relative binding affinities of these p53 cluster-sites, we propose a new p53 cluster-site algorithm that utilizes the trained PHMM to calculate and sum up the relative estimated binding-affinities, above a certain threshold, of all viable full-sites in the cluster with a spacer of ≤ 14 bp or less (see Methods). This model predicts a linear increase in p53 binding affinity dependent upon the number of half-sites in the cluster-site and the length of spacers between them. For example, for p53 cluster-sites with 2, 3, 4, 5, or 6 adjacent p53 half-sites, the number of possible full-sites with spacer-lengths = 14 bp would be 1, 3, 5, 7, and 9, respectively. Let N be the number of half-sites in the cluster-site, then the number of full-sites (to calculate binding affinities for and sum up) is given by the expression 2*N *- 3 (*N *≥ 2). Although there exist functional sites with spacers ≥ 15 bp, experiments suggest that their contribution to the overall binding affinity within a cluster-site is negligible.

**Table 1:** Normalized Experimental Affinity of Cluster-sites

	**Number of Half-sites**													
	**2**	**3**	**4**	**5**	**5.5**	**6**	**7**	**7.5**	**8**	**8.5**	**9**	**10**	**11**	**12**

**Cluster Site**	**Relative Binding Affinity**													

DDB2	1		5											

TP53I3		3			6			10		12			16	

**Theoretical Affinity Approximations**														

*# of Full-sites with spacers ≤ 14 bp*	*1*	*3*	*5*	*7*	*8*	*9*	*11*	*12*	*13*	*14*	*15*	*17*	*19*	*21*

# of Full-sites with spacers ≤ 24 bp	1	3	6	9	10.5	12	15	16.5	18	19.5	21	24	27	30

# of Full-sites with any size spacer	1	3	6	10		15	21		28		36	45	55	66

This table contains the normalized experimental affinities of different cluster-sites dependent upon the number of half-sites contained in the RE. These affinity measurements were obtained by mutating or truncating p53 cluster-sites in the genes DDB2, and TP53i3 [20,21]. These two p53 cluster-sites are chosen because they match the assumption of the theoretical models that no spacer sequences are present between the half-sites. All affinities are normalized by the 2 half-site (full-site) affinity respective of the RE. The theoretical models assume that all the half-sites in each cluster-site are identical, which is not the case for either of the two cluster-sites. Experimental results support a linear affinity growth model based upon the number of full-sites with spacers no longer than 14 bp (in italics).

These p53 cluster-site scores are attained through a two step process. The first step uses the cluster-site model which contains a generalized p53 half-site PHMM and a back-transition that limits any spacer between two half-sites to no more than 14 bp (see part e of Figure 2). The dynamic programming *Viterbi *algorithm is used to find the highest scoring p53 half-sites in the sequence (that are separated by no more then 14 bp). The second step then parses the state-path generated from step 1 and generates viable p53 full-sites with any spacers removed, while conserving the property that the half-sites in the cluster-site were not separated by more than 14 bp. Now we use the more flexible p53 single-site model to score these viable full-sites using the *Viterbi *algorithm (see part d of Figure 2). We maintain a running sum of the log-odds scores of the candidate full-sites that are above a certain threshold. The log-odds score threshold and spacer-length limit (14 bp) are chosen so as to best fit the experimental data (see Figure 7).

Additionally, this p53 cluster-site model follows statistical mechanics, in that the overall binding affinity for the complete RE is proportional to the probability of any p53 protein binding to any of the allowed motifs found in the cluster-site. (See Methods for more details.)

### Dynamic acceptance thresholds as a function of the distance from the TSS

**Figure 8:**
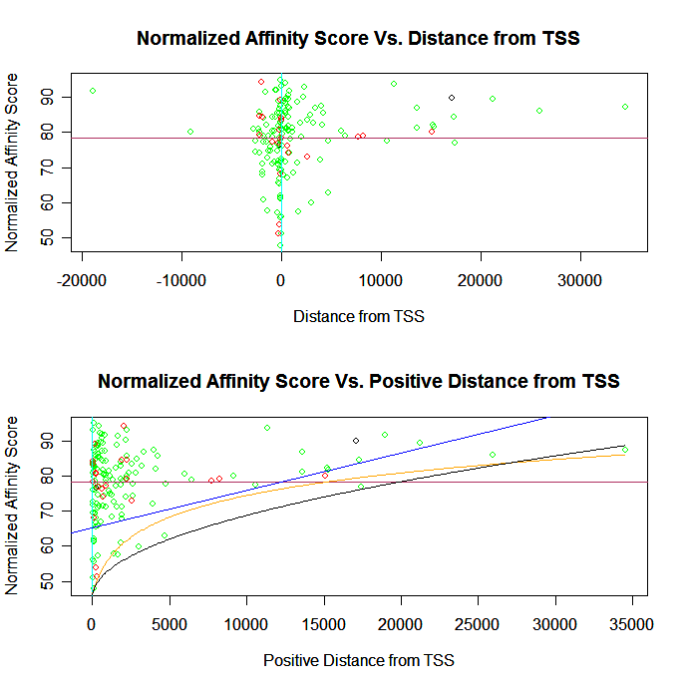
**Normalized affinity scores versus distances from the TSS**. **(Upper) **This plot presents the normalized affinity scores returned from the p53 single-site model versus the distance from the Transcription Start Site (TSS) for 158 experimentally validated p53-binding sites. Low affinity sites exist in a tight band around the TSS (cyan vertical line). p53 activation-sites are plotted in green, repression-sites in red, and both activation and repression in black. All sites ≥ 11 Kb from the TSS have relative affinity scores above the average of ≈ 78 (purple horizontal line). **(Lower) **This plot presents the estimated normalized affinity scores versus the positive distance (absolute value) from the TSS. Three dynamic acceptance thresholds are shown for scoring for putative p53 binding sites. The blue linear threshold corresponds to the formula .00107·Δ*X *+ 65.16 and has a false negative rate of 18 out of 158 validated p53 sites (11.4%). The orange logarithmic threshold corresponds to the formula 9.6854·log(Δ*X *+ 593.31) - 15.308 and has a false negative rate of 5 out of 158 validated p53 sites (3.2%). Finally, the black square-root threshold corresponds to the formula .23186·sqrt(Δ*X *+ 7.5231) + 45.6 and has a false negative rate of 1 out of 158 validated p53 sites (0.63%). (Δ*X *= distance from TSS)

An interesting finding from the analysis of our dataset of 160 functional p53 binding sites is that the low relative affinity scores from our model are significantly correlated with short distances from the Transcription Start Site (TSS). We find that low affinity sites exist only in a tight band around the TSS (see part a of Figure 8). Therefore a dynamic binding-affinity acceptance threshold, dependent upon the putative site's distance from the TSS, can greatly reduce the false positive rate of our classifier. With a dynamic acceptance threshold, putative sites will require higher calculated binding affinities as their distance from the TSS increases in order to be accepted as potentially functional. For example, consider the linear dynamic acceptance threshold .00107·Δ*X *+ 65.16 shown in Figure 8, with the additional restriction that the putative sites must be within 5,000 bp upstream and 1,000 bp downstream of the gene. Let the static acceptance threshold be all normalized scores above 70 with the same restriction that the putative sites must be within 5,000 bp upstream and 1,000 bp downstream of the gene. Even though the restricted dynamic threshold has a false negative rate of 22 out of 158 validated p53 sites (13.9%), and the restricted static threshold 32 out of 158 (20.3%), the restricted static threshold generates over 3.2 times as many positive hits when scoring all 39,288 isoforms of known genes in the human genome (hg18). Thus, the dynamic acceptance threshold has a lower known false negative rate and a considerably lower false positive rate. Different dynamic acceptance thresholds can be chosen to match desired levels of the known false negative rate and the genome hit rate (see part b of Figure 8). An important consideration when choosing an acceptance threshold is that a decrease in the threshold will in general produce an exponential increase in the number of positive hits.

## Conclusion

Profile Hidden Markov Models (PHMMs) can boost predictive power over weight matrices (PSSMs) when the binding motif is highly degenerative and tolerates insertions and/or deletions at various positions. The increase in predictive power for the p53-binding motif can be seen in Figures 4 and 5. When the RE has a known repeated and/or palindromic motif, this prior knowledge can be used to correspond parameters in the model to exploit the redundancy in the information in the motif. We propose a novel "Corresponded Baum-Welch" training algorithm that significantly boosts the predictive power of the p53-RE model, as seen in Figures 4 and 5. When the motif is not known, all possible motifs for the given size can be sampled and cross-validation techniques leveraged to infer the correct motif that maximizes predictive power. For example, Figure 5 reveals that the maximally predictive p53-binding motif corresponds the four quarter-sites in a combined-palindromic structure.

Our algorithms demonstrate the best predictive capability to date in classifying putative p53 binding sites. One algorithm uses a novel "Corresponded Baum-Welch" training method that exploits the repeated, palindromic structure of the p53 motif to train for allowed insertions and deletions relative to the consensus. The second algorithm properly models the relative increase in binding affinity for p53 cluster-sites (REs with ≥ 3 adjacent half-sites) by using a two step process that scores all viable full-sites in the cluster-site while restricting the spacer-length to 14 bp. This new cluster-site algorithm best matches the experimental data (see Figure 7).

Functional low-affinity p53-sites only exist near the TSS. Therefore the binding affinity threshold for accepting a putative site should be dependent on the putative site's distance from the TSS. By this method, putative sites with relatively low calculated binding affinities that are near the TSS may be accepted, while those sites with equal scores but more distant from the TSS will be rejected. A dynamic threshold, as a function of the distance from the TSS, can greatly reduce the false positive rate when searching for putative p53-sites in genes.

## Methods

### The Corresponded Baum-Welch algorithm

In order to exploit the redundancy of information in a homodimer or homotetramer binding motif, we wish to share information between corresponding positions. The level of sharing of information for any set of corresponding positions is given by a correspondence factor *c *such that 0 ≤ *c *≤ 1. At the end of each round of the iterative Baum-Welch algorithm we calculate the average values of each of the newly updated emission probabilities  and transition probabilities  for all *k *and *l *in the set of corresponding positions, represented as  and  respectively. Each of these average values represents the expected probability if the corresponding positions are fully tied (*c *= 1), and are referred to as the "corresponding average". Then we update the new emission and transition probabilities within the set of corresponding positions, using the current correspondence factor and corresponding average, according to:

(2)

If we wish to train for the optimum correspondence factor, then we calculate a new *c' *for each emission and transition probability at each position in the set of corresponding positions:

(3)

Now, we can calculate a new correspondence factor *c' *by averaging over sets of the  and  values. The one optimum correspondence factor for the whole motif or separate correspondence factors for sets of corresponding positions are obtained by averaging over different sets:

(4)

The Corresponded Baum-Welch algorithm will converge at (local) optimum emission and transition probabilities and correspondence factors that maximize the likelihood of observing the training set with possible pseudo-counts. Please see the Additional file 1 for further details.

### The p53 cluster-site algorithm

The p53 cluster-site algorithm is a two step process designed to sum the estimated relative binding affinities of all viable full-sites within a cluster-site. The first step uses the cluster-site model that contains a generalized p53 half-site PHMM and a back-transition through a no-cost FEM-14 module (see part e of Figure 2). The no-cost Finite Emission Module (FEM) of length 14 can match any sequence of length ≤ 14 bp with no contribution to the over-all score. We score the entire putative cluster-site using the p53 cluster-site model and the *Viterbi *algorithm to find the best-supported path through the cluster-site. This path provides the strongest affinity half-sites that are not separated by more than 14 bp. If we use the notation "14" for any spacer sequence of length 0 to 14 and *H *for a half-site sequence, then we can represent the cluster-site sequence path as:



Step 2 now parses the cluster-site sequence path and generates a list of all viable full-sites, which are concatenations of any two half-sites such that they are not separated by more than 14 bp:



Now we use the more flexible (and more accurate) single-site model with the Viterbi algorithm to estimate the relative binding affinity of all the viable full-sites in the cluster-site. The cluster-site affinity score is the sum of all viable full-site scores that exceed a certain threshold. If *F *denotes a viable full-site then:

(5)

The spacer-length upper bound and the affinity-score lower bound were fit to best match the experimental results. In the case for p53-binding sites, the best fit is a spacer-length of no more than 14 bp and a log-odds score of at least 27.5 (see Figure 7).

### The p53HMM implementation

The p53HMM algorithm is implemented in Java and is available on-line at . The implementation makes extensive use of the BioJava Toolkit [24].

## Authors' contributions

TR participated in the design of the algorithms, wrote the code, performed the computational analysis, and drafted the manuscript. XY performed all experiments. ES participated in the design of the algorithms and helped to draft the manuscript. AL conceived of the study and helped to draft the manuscript. All authors read, edited, and approved the final version of the paper.

## Supplementary Material

Additional File 1 **Supplementary Material**. The Supplementary Material contains more theory behind modeling TF-binding sites with PHMMs, details of the Corresponded Baum-Welch algorithm, and a proof that the PHMM log-odds score of a TF-binding site estimates its relative binding affinity given certain assumptions. Click here for file
