# Supplementary material for: The p53HMM algorithm: using profile hidden markov models to detect p53-responsive genes
Source: BMC Bioinformatics. 2009 Apr 20;10:111. doi: 10.1186/1471-2105-10-111 (PMC2685388; doi:10.1186/1471-2105-10-111)
Supplement: Additional File 1 — Supplementary Material. The Supplementary Material contains more theory behind modeling TF-binding sites with PHMMs, details of the Corresponded Baum-Welch algorithm, and a proof that the PHMM log-odds score of a TF-binding site estimates its relative binding affinity given certain assumptions. [file 1471-2105-10-111-S1.pdf]

The p53HMM algorithm: using profile hidden markov models to detect p53-responsive genes

Todd Riley*[1,2] , Xin Yu[4] , Eduardo Sontag[2,3] and Arnold Levine[1,4]

[1]The Institute for Advanced Study, Princeton, New Jersey
[2]The BioMaPS Institute at Rutgers University, Piscataway, New Jersey
[3]The Mathematics Department, Rutgers University, Piscataway, New Jersey
[4]The Cancer Institute of New Jersey, New Brunswick, New Jersey

Email: TR: tr2261@columbia.edu; XY: yuxi@umdnj.edu; ES: sontag@math.rutgers.edu; AL: alevine@ias.edu;

*Corresponding author

Supplementary Material

The Theory of Modeling TF-Binding Sites with Profile Hidden Markov Models

Given a set S of experimentally validated binding sites s for a TF-protein (and a few assumptions) it is possible to use the set S to estimate the relative binding free energy $-\Delta G(x)$ of any putative site x (without having to perform direct experimental measurements of binding constants). This bioinformatic approach using PHMMs (and PSSMs) is an attractive alternative, if a sufficient set S of experimentally validated binding sites is available.

The Assumptions:

1. The positions of a binding site contribute independently and additively to the binding free-energy
2. Background DNA sequences are generally random samples from some k-mer distribution

Neither of these assumptions are always true [1]. The first assumption can be relaxed by calculating di-nucleotide, tri-nucleotide,....,n^{th}-nucleotide frequencies from the training set S, but at some point an additivity assumption must be applied. Also, genomes are generally not random, but can be closely approximated by a 3rd or 4th Order Markov Model [2]. For simplicity in the examples here, we will assume that the background DNA can be modeled by a simple 0th Order Markov Model (i.e. by mononucleotide content alone). This assumption greatly simplifies the calculation of the partition function [1].

From the additivity assumption we have that for any putative site x:

$$-\Delta G(x) = \sum_{i=1}^{length(x)} -\Delta G_j(b)$$

where we define...

$$\begin{aligned} -\Delta G_j(b) &= \text{the independent contribution of base } b \text{ observed at position } j \\ &\quad \text{to the over-all binding free energy} \end{aligned} \qquad (1)$$

The Profile Hidden Markov Model (PHMM) provides a completely probabilistic model for observing a sequence x within the modeled motif. The PHMM achieves this by incorporating the probabilities of different nucleotide insertions, deletions, and motif matches at each position in the motif [3]. In this application, the PHMM model is used to calculate the probability $P_{hmm}(x)$ of observing the putative site x in a real transcription factor binding site that is modeled by the PHMM. The probability $P_{hmm}(x)$ is used to find the *site log-odds score* of a putative site x. The *site log-odds score* $G^s(x)$ calculated by a PHMM trained by S is given by:

$$\begin{aligned} G^s(x) &= \log_e\left(\frac{P_{hmm}(x)}{P_{background}(x)}\right) \qquad \text{(Site Log-odds Score)} \\ &= \sum_{j=1}^{\text{length}(x)} G^s_j(b) \end{aligned}$$

where we define:

$$\begin{aligned} G^s_j(b) &= \log_e\left(\frac{P_{hmm}(j,b)}{P_{background}(j,b)}\right) \qquad \text{(Nucleotide Log-odds Score)} \\ j &= \text{position in the sequence } x,\ j \in \{1 \dots \text{ length}(x)\} \\ b &= \text{observed nucleotide base, } b \in \{A, C, G, T\} \\ P_{hmm}(j,b) &= \text{probability of base } b \text{ at position } j \text{ in the PHMM model} \\ P_{background}(j,b) &= \text{probability of base } b \text{ at position } j \text{ in the null (background) model} \end{aligned} \qquad (2)$$

With these definitions, and assuming independence of positions, we have:

$$\begin{aligned} P_{hmm}(x) &= \text{probability of candidate site } x \text{ in the PHMM model} \\ P_{background}(x) &= \text{probability of candidate site } x \text{ in the null (background) model} \end{aligned}$$

The *Site Log-odds Score* $G^s(x)$ can be considered proportional to the relative binding free energy $-\Delta G(x)$ when the Fermi-Dirac Equation for the equilibrium probability of a protein-bound binding site can be

approximated by the Maxwell-Boltzmann Equation [4]. Another assumption is that the training set S consists of a proper sampling of functional binding sites that were collected under similar experimental conditions (like temperature T). However, this is likely not the case. A last assumption is that we are able to perfectly train the PHMM from our training set S, so that we can accurately predict the probability $P_{hmm}(x)$ for all possible putative sites x. However, properly training a PHMM from a limited training set S is a challenging problem. But with our idealizations and assumptions, the *Nucleotide Log-odds Score* $G^s_j(b)$ (calculated by our perfectly trained PHMM) is directly proportional to the binding free energy contribution of each observed base b at each position j in the sequence x.

Thus, under ideal conditions the log-odds scores that a trained Profile Hidden Markov Model calculates for any candidate site x is directly proportional to the free energy of binding to that candidate site. (Typically, proper scaling of G^s if not performed to make $G^s(x) \approx -\Delta G(x)$. Instead, G^s is only proportional to $-\Delta G(x)$.) [5] If the Profile Hidden Markov Model has no insertion or deletion states, then the PHMM is essentially a PSSM (weight matrix), and the probability $P_{hmm}(j, b)$ is equivalent to the $(b, j)^{th}$ entry in the (probability) weight matrix.

Three dynamic programming algorithms are used to calculate the probability $P_{hmm}(x)$ of observing the putative site x in the model. The *forward* and *backward* algorithms calculate $P_{hmm}(x)$ by summing up the probability of observing x for all possible paths π through the model:

$$forward(x) = backward(x) = P_{hmm}(x) = \sum_{\pi}^{all\ paths} P(x, \pi) \tag{3}$$

The *Viterbi* algorithm calculates both the optimal alignment of the putative site x which produces the path $\pi^*(x)$ with the highest log-odds score, and the probability $P^{\pi^*}_{hmm}(x)$ of observing that optimal path in the model. These two results of the Viterbi algorithm are commonly referred to as the *Viterbi path* and the *Viterbi score*, respectively:

$$\begin{aligned} Viterbi\ path(x) &= \pi^*(x) &= \underset{\pi}{\text{argmax}}\,[P(x, \pi)] \\ Viterbi\ score(x) &= P^{\pi^*}_{hmm}(x) &= P_{hmm}(x, \pi^*(x)) \end{aligned}$$

In the case of modeling transcription factor binding sites, it is commonly assumed that the log-odds score of the optimal path that best aligns the putative site x to the model is the only significant contributor to the over-all log-odds score. When this is indeed true, the *Viterbi score* can be used as a good

approximation to $P_{hmm}(x)$:

$$Viterbi\ score(x) \quad = \quad P_{hmm}(x, \pi^*(x)) \quad \approx \quad \sum_{\pi}^{all\ paths} P(x, \pi) \quad = \quad P_{hmm}(x) \quad = \quad forward(x) \qquad (4)$$

However, we see that this assumption is not true when modeling p53 cluster sites, where experiments suggest that the p53 protein can bind to overlapping combinations of adjacent half-sites. In this scenario, the true probability $P_{hmm}(x)$ provided by the *forward* and *backward* algorithms is needed to properly model experimental results.

All three dynamic programming algorithms are highly efficient, and when applied to PHMMs run in $O(NM)$ time and $O(NM)$ space for a PHMM with M states and a sequence of length N [6]. For further details about the *forward*, *backward*, and *Viterbi* algorithms please see [5].

The Corresponded Baum-Welch Algorithm

The standard Baum-Welch EM algorithm is used to estimate the expected transition and emission probabilities from the training set. The Baum-Welch algorithm is an optimized, iterative EM method that always climbs the gradient and uses the dynamic programming *forward* and *backward* algorithms [5].

Let:

s	$=$	*binding site*	The nucleotide sequence of a binding site
s_i	$=$	*nucleotide*	The ith nucleotide in the binding site s
S	$=$	*training set*	The training set of binding sites s_j
π	$=$	*path*	The state sequence of a binding site s
π_i	$=$	*state*	The ith state in the path π
ps_{kl}	$=$	*pseudocount*	Prior bias of probability of transition from k to l
$ps_k(b)$	$=$	*pseudocount*	Prior bias of probability of emitting symbol b in state k
ψ	$=$	$\{ps_{kl}, ps_k(b)\}, \forall k, l, b$	The set of all pseudocounts in the model
a_{kl}	$=$	$P(\pi_i = l \vert \pi_{i-1} = k)$	The probability of transition from state k to state l
$e_k(b)$	$=$	$P(s_i = b \vert pi_i = k)$	The probability of emitting symbol b in state k
θ	$=$	$\{a_{kl}, e_k(b)\}, \forall k, l, b$	The set of all parameters in the model
$a_{kl}^{background}$	$=$	$P_{background}(\pi_i = l \vert \pi_{i-1} = k)$	The probability of transition from state k to state l in the null (background) model
$e_k^{background}(b)$	$=$	$P_{background}(s_i = b \vert pi_i = k)$	The probability of emitting symbol b in state k in the null (background) model
A_{kl}	$=$	*expected* a_{kl} *counts*	Number of transitions from k to l in the training set
$E_k(b)$	$=$	*expected* $e_k(b)$ *counts*	Number of emissions of b from state k in the training set
$f_k(i)$	$=$	$P(s_1 \dots s_i, \pi_i = k)$	The probability of the sequence up to and including s_i, requiring that $\pi_i = k$
$f_k(i+1)$	$=$	$e_k(s_{i+1}) \cdot \sum_j^{states}(f_j(i) \cdot a_{jk})$	Recursive formula for $f_k(i+1)$ going forward
$b_k(i)$	$=$	$P(s_i \dots s_L, \pi_i = k)$	The probability of the sequence from s_i to the end, requiring that $\pi_i = k$, $L =$ length of the sequence s
$b_k(i-1)$	$=$	$e_k(s_{i-1}) \cdot \sum_j^{states}(b_j(i) \cdot a_{jk})$	Recursive formula for $b_k(i-1)$ going backward

The goal is to choose the parameters θ of the model in order to maximize the log-likelihood of the sequences s in the training set S, without over-fitting. To avoid over-fitting, the goal is to find the Posterior Mean Estimator (PME), a Bayesian approach that uses the pseudo-counts ψ as a prior from a Dirichlet family of distributions and all the paths π for all sequences s in the training set S [5]:

$$\theta^{PME} = \underset{\theta}{\text{argmax}} \left[\sum_{s \in S} \log P(s|\theta, \psi) \right] = \underset{\theta}{\text{argmax}} \left[\sum_{s \in S} \sum_{\pi} \log P(s, \pi|\theta, \psi) \right]$$

The Baum-Welch algorithm climbs the gradient during each iteration and is guaranteed to converge within some epsilon to a local maximum, which may or may not be the PME [5]. Theoretically, the Corresponded Baum-Welch algorithm has the advantage of using prior motif knowledge to greatly reduce the parameter space and to potentially "flatten" the space. Both of these improvements can increase the probability of the algorithm converging to the PME.

In each iteration, the Baum-Welch algorithm calculates the expected number of times each transition and emission is used by the training set sequences (calculates A_{kl} and $E_k(b)$), given the current model parameters (a_{kl} and $e_k(b)$). Then the model parameters are updated to the new posterior mean estimators

a'_{kl} and $e'_k(b)$, calculated from the new expectation counts (A_{kl} and $E_k(b)$).

Notice that the probability that a_{kl} is used at position i of binding site sequence s with current model parameters θ is given by:

$$P(\pi_i = k, \pi_{i+1} = l|s, \theta) = \frac{f_k(i) \cdot a_{kl} \cdot e_l(s_{i+1}) \cdot b_l(i+1)}{P(s)}$$

By summing over all training sequences and positions, we can derive A_{kl} and $E_k(b)$, the expected number of times that a_{kl} and $e_k(b)$ are used by the training set, given the current model parameters θ:

$$\begin{aligned}
N &= \text{ number of training sequences} \\
L &= \text{ length of the sequence } s^j \\
W(s^j) &= \text{ sequence weight of } s^j \\
A_{kl} &= \sum_{s^j \in S}^{N} \frac{W(s^j)}{P(s^j)} \sum_{i=1}^{L} f_k^j(i) \cdot a_{kl} \cdot e_l(s_{i+1}^j) \cdot b_l^j(i+1) \\
E_k(b) &= \sum_{s^j \in S}^{N} \frac{W(s^j)}{P(s^j)} \sum_{i|s_i^j = b}^{L} f_k^j(i) \cdot b_k^j(i)
\end{aligned} \tag{5}$$

The sequence weight $W(s^j)$ is used to vary the importance of different sequences in the training set S and to vary their influence in training the model. A weight $W(s^j) > 1$ increases the expected counts in sequence s^j, and a weight $W(s^j) < 1$ decreases them. Sequence weights are used when we do not fully trust that the training set S provides a proper distribution of valid binding sites, and we attempt to remedy that deficiency by weighting the known sequences. Most sequence weighting methods attempt to penalize the expected counts of similar sequences and to enhance the expected counts of distant sequences [5].

Additionally, the process by which the training set S was ascertained may be biased toward a certain subset of sites independent of their sequences (*ascertainment bias*). In the derivation for our approximation for $-\Delta G(x)$ in the next section, we relied on the assumption that the probability $P_{extract}(x)$ of extracting a TF-bound binding site was independent of the sequence in or around x. This may not always be the case. For example, if we know that a certain antibody preferentially binds to adjacent binding sites compared to ones with no neighbors, then after precipitation our training set S would be biased toward adjacent binding sites that appear in tight clusters in the DNA. We could attempt to compensate for this inherent bias by penalizing those sequences found adjacent to each other in the genome and promoting the

ones with no neighbors. Different sequence weighting schemes can be found in [7–13].

From these new expected counts, we can now calculate new maximum likelihood estimators for each position:

$$
\begin{aligned}
a'_{kl} &= \frac{A_{kl}}{\sum_{m}^{states} A_{km}} \\
e'_k(b) &= \frac{E_k(b)}{\sum_{n}^{\{A,C,G,T\}} E_k(n)}
\end{aligned}
\tag{6}
$$

However, if we believe the training set S to be incomplete and intend to avoid over-fitting the data, we add pseudocounts as priors to our expected counts. Here, pseudocounts are distributed in proportion to the null (background) model. The pseudocount weight w represents how many counts from the null (background) model we want to include in the expected counts of our model. From the expected counts, we calculate the new posterior mean estimators using pseudocounts for each position:

$$
\begin{aligned}
w &= \text{pseudocount weight} \\
ps_{kl} &= w \cdot a_{kl}^{background} \\
ps_k(b) &= w \cdot e_k^{background}(b) \\
a'_{kl} &= \frac{ps_{kl} + A_{kl}}{w + \sum_{m}^{states} A_{km}} \\
e'_k(b) &= \frac{ps_k(b) + E_k(b)}{w + \sum_{n}^{\{A,C,G,T\}} E_k(n)}
\end{aligned}
\tag{7}
$$

Now we use the prior knowledge (or make a guess) of the repeat and/or palindromic motif and correspond (partially or fully tie) the new posterior mean estimators based upon corresponding positions. This prior knowledge can be used to reduce the parameter space and increase the statistical accuracy of the model. The degree of sharing of information between corresponding positions is controlled by a correspondence factor c, which can be fixed or trained to an optimum value. One can estimate a correspondence factor

based on the initial conditions by the following:

$$\begin{aligned}
dist &= \text{a probability distribution in the set of corresponding distributions} \\
var &= \text{a variable in the probability distributions} \\
N &= \text{number of corresponding distributions} \\
\overline{P(var)} &= \text{average probability of a variable over all corresponding distributions} \\
c_0 &= \text{initial correspondence factor} \\
&= 1 \quad - \quad \frac{1}{N-1} \sum_{dist} \sum_{var} \left| \overline{P(var)} - P(var) \right| \qquad (8)
\end{aligned}$$

We calculate the corresponding posterior mean estimator (PME) after calculating the average emission and transition probabilities for all the corresponding positions:

$$\begin{aligned}
c &= \text{correspondence factor} \\
\overline{a'} &= Avg(a'_{kl}) \quad \text{(over all transitions from } k \text{ to } l \text{ in the set of corresponding positions)} \\
\overline{e'(b)} &= Avg(e'_k(b)) \quad \text{(over all emissions in the set of corresponding positions)} \\
a''_{kl} &= a'_{kl} + c \left[\overline{a'} - a'_{kl} \right] \\
e''_k(b) &= e'_k(b) + c \left[\overline{e'(b)} - e'_k(b) \right] \qquad (9)
\end{aligned}$$

If we wish to train for the optimum correspondence factor, then we calculate a new c' for each emission and transition probability at each position in the set of corresponding positions:

$$\begin{aligned}
c'_{kl} &= \frac{c \cdot \overline{a'}}{a'_{kl} + c \left[\overline{a'} - a'_{kl} \right]} = \frac{c \cdot \overline{a'}}{a''_{kl}} \\
c'_k(b) &= \frac{c \cdot \overline{e'(b)}}{e'_k(b) + c \left[\overline{e'(b)} - e'_k(b) \right]} = \frac{c \cdot \overline{e'(b)}}{e''_k(b)} \qquad (10)
\end{aligned}$$

Now, we can calculate a new correspondence factor c' by averaging over sets of the c'_{kl} and $c'_k(b)$ values. The one optimum correspondence factor for the whole motif or separate correspondence factors for sets of corresponding positions are obtained by averaging over different sets:

$$c' = \overline{c'_k(b)} \quad \text{(over all bases } b \text{ and all emissions and transitions } k\text{)}$$

or

$$\text{(over all bases } b \text{ and corresponding emissions and transitions } k\text{)} \qquad (11)$$

We can now update the parameters of the model to the new posterior mean estimators that have been made corresponding (fully or partially tied) by our prior knowledge (or guess) of the motif:

$$\begin{aligned} a_{kl} &\Longrightarrow a''_{kl} \\ e_k(b) &\Longrightarrow e''_k(b) \\ c &\Longrightarrow c' \end{aligned} \tag{12}$$

This process is then iterated to obtain new A_{kl} and $E_k(b)$ values from the new model parameters. At each iteration the log likelihood of the training set increases to a local maximum. Since convergence is in a continuous-valued space, the maximum is never actually reached. Typically, the iterations are stopped when the change in the total log likelihood is sufficiently small or after some fixed number of iterations, whichever comes first [5].

Derivation of finding optimum correspondence. The method of finding the locally optimum degree of correspondence (sharing of information) between corresponding positions starts by introducing the new parameter c for each set of corresponding positions. If we interpret the correspondence factor c as the probability $P(\text{identical})$ that the positions are completely synonymous, then we can interpret that every emission and transition probability $P(x)$ for each corresponding position in the model can now be replaced by a new probability $P'(x)$:

$$\begin{aligned} P'(x) &= P(\text{identical}) \cdot \overline{P(x)} \quad + \quad (1 - P(\text{identical})) \cdot P(x) \\ &= P(x) + c\left[\overline{P(x)} - P(x)\right] \end{aligned} \tag{13}$$

where $\overline{P(x)}$ is the average of the corresponding emission and transition probabilities. Now we can calculate new correspondence factors c' for each corresponding emission and transition probability in the set of corresponding positions:

$$\begin{aligned} c' &= \frac{P(\text{identical}) \cdot \overline{P(x)}}{P(\text{identical}) \cdot \overline{P(x)} \quad + \quad (1 - P(\text{identical})) \cdot P(x)} \\ &= \frac{c \cdot \overline{P(x)}}{P(x) + c\left[\overline{P(x)} - P(x)\right]} \\ &= \frac{c \cdot \overline{P(x)}}{P'(x)} \end{aligned} \tag{14}$$

Now we can calculate a new correspondence factor c'' for the set of corresponding parameters by averaging over the new c' for all the corresponding emission and transition probabilities:

$$c'' = \overline{c'} \quad \text{(over all } c' \text{ in the set of corresponding positions)} \tag{15}$$

Example. Assume that we have prior knowledge (or we guess that) the binding motif of a 10-bp binding site is singly palindromic: *1 2 3 4 5 5 4 3 2 1*. Then the positions that have been made corresponding are: *1* and *10*, *2* and *9*, *3* and *8*, *4* and *7*, *5* and *6*. (There are five sets of corresponding positions in this example.) First, each of the 10 distributions of the posterior mean emission probabilities for each of the 10 positions in the motif are now corresponding and sharing data with its partner position. Then the posterior mean transition distributions between positions are similarly made corresponding (for example *1-2* and *2-1*). Separate correspondence calculations are performed for each of the sets of corresponding positions. A correspondence factor of $c = 1$ would fully correspond (tie) the parameters between synonymous positions to the average over all corresponding parameters. (In this case, the parameter space would roughly be cut in half, and the training data per parameter would roughly double.) A correspondence factor of $c = 0$ would not change the initial distributions of emission and transition probabilities at a position at all, thus creating no correspondence between the positions. The correspondence factor c can be regarded as our *known* prior belief in the level of correspondence between synonymous positions in a palindromic, repeat, and/or reverse-complement binding-site motif. Alternatively, the correspondence factor c can be regarded as the *unknown* probability of correspondence between synonymous positions that needs to be determined. In the latter case, the Corresponded Baum-Welch algorithm will converge on the (locally) optimum c that maximizes the total log likelihood of the training set.

The Proof that the Log-odds Score $G^s(x)$ is proportional to $-\Delta G(x)$

It has been shown experimentally that in general, transcription factor proteins have a weak affinity for background DNA (any non-consensus sequence) and a strong affinity for consensus sites. Within the nucleus (or general cell in prokaryotes) the DNA concentration is high enough that an activated TF-protein is bound somewhere on the DNA essentially all the time (to a 1st approximation) [14]. Therefore, the binding specificity (the ability of the TF protein to distinguish a functional site from background DNA) must be adequately high for proper regulation to occur [14]. The goal is to quantify the free energy of binding to a candidate site x through statistical mechanics, thermodynamics and Information Theory. We

start with the mass action kinetics of a TF-protein binding to a site:

$$\begin{aligned} p &= \text{transcription factor protein} \\ x &= \text{a candidate DNA binding site} \\ px &= \text{Bound Protein-Binding Site Complex} \\ k^+ &= \text{forward equilibrium binding constant} \\ k^- &= \text{backward equilibrium binding constant} \\ p + x &\underset{k^-}{\overset{k^+}{\rightleftharpoons}} px \\ K_{eq}^x &= \frac{k^+}{k^-} = \text{equilibrium association constant for site } x \end{aligned} \quad (16)$$

We normalize K_{eq}^x in order to obtain the specific association constant K_s^x that quantifies specificity:

1. K_{eq}^{avg} = Average K_{eq} for all sites x
2. $K_s^x = \frac{K_{eq}^x}{K_{eq}^{avg}}, \quad (avg(K_s^x) = 1)$
3. Specificity of Valid Site: $K_s^{\text{valid site}} \approx 10^6$
4. Specificity of Background: $K_s^{\text{background}} < 1$

In experiments performed in E. Coli cells, with about 5×10^6bp of DNA, a single TF-protein and a single binding site with a specificity of 10^6 will be bound together only about 20% of the time. During the other 80% of the time, the protein will be transiently bound to other random places along the genome. However, with 20 copies of the protein the binding site will be occupied about 99% of the time [15].

The specific association constant K_s^x is related to the binding free energy $-\Delta G(x)$ by the following:

$$-\Delta G(x) = -k_\beta \cdot T \cdot \ln(K_s^x)$$

and

$$-K_s^x = \frac{k^+}{k^- \cdot K_{eq}^{avg}} = e^{-\Delta G(x)/k_\beta T} \quad (17)$$

Now lets estimate the probability that a putative binding site x is bound by a TF-protein in a well-mixed solution at equilibrium. Let $P(x \text{ bound})$ be the probability that the binding site x is bound by a

TF-protein. Then we have:

$$\begin{aligned} P(x \text{ bound}) &= \frac{\text{binding rate}}{\text{binding rate} + \text{unbinding rate}} \\ &= \frac{[p]\cdot k^{+}}{[p]\cdot k^{+} + k^{-}} \\ &= \frac{[p]\cdot K_{eq}^{avg}\cdot e^{-\Delta G(x)/k_\beta T}}{[p]\cdot K_{eq}^{avg}\cdot e^{-\Delta G(x)/k_\beta T} + 1} \end{aligned} \tag{18}$$

which can be re-written into the form known as the Fermi-Dirac Equation, where $\mu = k_\beta T \ln(K_{eq}^{avg}\cdot [p])$ is the *chemical potential* dependent on the protein concentration $[p]$:

$$P(x \text{ bound}) = \frac{1}{e^{(\Delta G(x)-\mu)/k_\beta T} + 1} \qquad (Fermi - Dirac)$$

In the low concentration limit the Fermi-Dirac Equation for the probability $P(x \text{ bound})$ can be approximated by the Maxwell-Boltzmann Equation:

$$\begin{aligned} P(x \text{ bound}) &\approx \frac{1}{e^{(\Delta G(x)-\mu)/k_\beta T}} \quad \text{when } \Delta G(x) \gg \mu \\ &\approx e^{\mu/k_\beta T}\cdot e^{-\Delta G(x)/k_\beta T} \qquad (Maxwell - Boltzmann) \\ &\approx z e^{-\Delta G(x)/k_\beta T} \qquad (z = e^{\mu/k_\beta T} = \text{fugacity}) \end{aligned} \tag{19}$$

Now we are ready to analyze a sampling set S of known transcription factor binding sites for a given TF-protein. A version of this proof exists for weight matrices (PSSMs) in [4,16]. Here we provide a general proof that it is applicable for any fully probabilistic model that calculates $P_{background}(x)$ and $P_{setS}(x)$.

Assume that we attain the set S from a single experiment so that all the sites are collected under identical conditions. Assume that we have a very large number of DNA sequences of roughly similar length from a given genome mixed in solution with a certain concentration of TF-proteins. At equilibrium some of the DNA sequences with bound TF-protein are extracted (precipitated) and sequenced to create our sampling set S.

The probability of observing exactly the set S is given by:

$$\begin{aligned} P(\text{observing the set } S) &= \prod_{x\in S}(P_{exist}(x)\cdot P_{bound}(x)\cdot P_{extract}(x))\cdot \prod_{x\notin S}(1 - P_{exist}(x)\cdot P_{bound}(x)\cdot P_{extract}(x)) \\ &\approx \prod_{x\in S}(P_{exist}(x)\cdot P_{bound}(x)\cdot P_{extract}(x))\cdot e^{\sum_{x\notin S}(P_{exist}(x)\cdot P_{bound}(x)\cdot P_{extract}(x))} \end{aligned} \tag{20}$$

The likelihood function $\mathscr{L}$ for the P(observing the set S) can now be approximated:

$$\begin{aligned}
\mathscr{L} &= \ln\left[P(\text{observing the set } S)\right] \\
&\approx \ln\left[\prod_{x\in S}\left(P_{exist}(x)\cdot P_{bound}(x)\cdot P_{extract}(x)\right)\cdot e^{\sum_{x\notin S}(P_{exist}(x)\cdot P_{bound}(x)\cdot P_{extract}(x))}\right] \\
&\approx \sum_{x\in S}\ln\left(P_{exist}(x)\cdot P_{bound}(x)\cdot P_{extract}(x)\right) - \sum_{x\notin S}\left(P_{exist}(x)\cdot P_{bound}(x)\cdot P_{extract}(x)\right) \qquad (21)
\end{aligned}$$

Now plug-in the Maxwell-Boltzmann approximation $ze^{-\Delta G(x)/k_\beta T}$ for $P(x$ bound), and for simplicity assume that $P_{extract}(x) = P_{extract}$ is identical for all x:

$$\begin{aligned}
\mathscr{L} &\approx \sum_{x\in S}\ln\left(P_{exist}(x)\cdot ze^{-\Delta G(x)/k_\beta T}\cdot P_{extract}\right) - \sum_{x\notin S}\left(P_{exist}(x)\cdot ze^{-\Delta G(x)/k_\beta T}\cdot P_{extract}\right) \\
&\approx N_s\cdot\ln(z\cdot P_{extract}) + \sum_{x\in S}\left(\ln(P_{exist}(x))\cdot\frac{-\Delta G(x)}{k_\beta T}\right) - z\cdot P_{extract}\sum_{x\notin S}\left(P_{exist}(x)\cdot e^{-\Delta G(x)/k_\beta T}\right)
\end{aligned} \qquad (22)$$

Where N_s is the size of the sampling set S. We are now ready to maximize the likelihood function $\mathscr{L}$ by taking the partial derivatives with respect to $zP_{extract}$ and $\Delta G_i(b)$ and setting them equal to 0. We have From the additivity assumption that for any putative site x:

$$-\Delta G(x) = \sum_{i=1}^{length(x)} -\Delta G_i(b)$$

where we define...

$$\begin{aligned}
-\Delta G_i(b) &= \text{the independent contribution of base } b \text{ observed at position } i \\
-\Delta G_i(x,b) &= -\Delta G_i(b)\cdot x(i,b) \\
x(i,b) &= 1 \text{ if } x_i = b\text{, and } 0 \text{ if } x_i \neq b \qquad (23)
\end{aligned}$$

After taking the partial derivatives we have:

$$\begin{aligned}
\frac{\partial\mathscr{L}}{\partial(zP_{extract})} &= \frac{N_s}{z\cdot P_{extract}} - \sum_{x\notin S}\left(P_{exist}(x)\cdot e^{-\Delta G(x)/k_\beta T}\right) = 0 \\
\frac{\partial\mathscr{L}}{\partial(\Delta G_i(b))} &= \frac{\sum_{x\in S}x(i,b)}{k_\beta T} - \left[\frac{z\cdot P_{extract}}{k_\beta T}\cdot P_{exists}(i,b)\cdot e^{-\Delta G_i(b)/k_\beta T}\cdot\prod_{j\neq i}\sum_{b'}P_{exists}(j,b')\cdot e^{-\Delta G_j(b')/k_\beta T}\right] = 0
\end{aligned} \qquad (24)$$

We can combine the results from the partial derivatives to obtain:

$$\frac{1}{N_s}\sum_{x\in S}x(i,b) = \frac{P_{exists}(i,b)\cdot e^{-\Delta G_i(b)/k_\beta T}\cdot\prod_{j\neq i}\sum_{b'}P_{exists}(j,b')\cdot e^{-\Delta G_j(b')/k_\beta T}}{\sum_{x\notin S}\left(P_{exist}(x)\cdot e^{-\Delta G(x)/k_\beta T}\right)} \qquad (25)$$

If we make the observation that:

$$\sum_{x \notin S} \left(P_{exist}(x) \cdot e^{-\Delta G(x)/k_\beta T} \right) = \sum_{b'} P_{exists}(i,b') \cdot e^{-\Delta G_i(b')/k_\beta T} \cdot \prod_{j \neq i} \sum_{b'} P_{exists}(j,b') \cdot e^{-\Delta G_j(b')/k_\beta T}$$

then we have that:

$$\begin{aligned}
\frac{1}{N_s} \sum_{x \in S} x(i,b) &= \frac{P_{exists}(i,b) \cdot e^{-\Delta G_i(b)/k_\beta T} \cdot \prod_{j \neq i} \sum_{b'} P_{exists}(j,b') \cdot e^{-\Delta G_j(b')/k_\beta T}}{\sum_{b'} P_{exists}(i,b') \cdot e^{-\Delta G_i(b')/k_\beta T} \cdot \prod_{j \neq i} \sum_{b'} P_{exists}(j,b') \cdot e^{-\Delta G_j(b')/k_\beta T}} \\
&= \frac{P_{exists}(i,b) \cdot e^{-\Delta G_i(b)/k_\beta T}}{\sum_{b'} P_{exists}(i,b') \cdot e^{-\Delta G_i(b')/k_\beta T}} \\
&= \frac{P_{exists}(i,b) \cdot e^{-\Delta G_i(b)/k_\beta T}}{C} \\
\frac{\frac{1}{N_s} \sum_{x \in S} x(i,b)}{P_{exists}(i,b)} \cdot C &= e^{-\Delta G_i(b)/k_\beta T} \\
\ln \left[\frac{\frac{1}{N_s} \sum_{x \in S} x(i,b)}{P_{exists}(i,b)} \right] + \ln C &= -\frac{\Delta G_i(b)}{k_\beta T} \\
\ln \left[\frac{\frac{1}{N_s} \sum_{x \in S} x(i,b)}{P_{exists}(i,b)} \right] &\approx\propto -\Delta G_i(b) \qquad (26)
\end{aligned}$$

Now we make the following observations:

$$\begin{aligned}
\frac{1}{N_s} \sum_{x \in S} x(i,b) &= \text{probability of observing base b at position i in our set } S \\
&= P_{setS}(x_i(b)) \\
P_{exists}(i,b) &= P_{background}(i,b) \qquad (27)
\end{aligned}$$

So now we have:

$$\begin{aligned}
G_i^s(b) &= \ln \left[\frac{P_{setS}(x_i(b))}{P_{background}(i,b)} \right] \approx\propto -\Delta G_i(b) \\
G^s(x) &= \ln \left[\frac{P_{setS}(x)}{P_{background}(x)} \right] \approx\propto -\Delta G(x) \qquad \text{(by the additivity assumption)}
\end{aligned}$$

□

References

1. Stormo GD: **DNA binding sites: representation and discovery**. *Bioinformatics* 2000, **16**:16–23, [http://bioinformatics.oxfordjournals.org/cgi/content/abstract/16/1/16].
2. Thijs G, Lescot M, Marchal K, Rombauts S, De Moor B, Rouze P, Moreau Y: **A higher-order background model improves the detection of promoter regulatory elements by Gibbs sampling**. *Bioinformatics* December 2001, **17**:1113–1122(10), [http://www.ingentaconnect.com/content/oup/cabios/2001/00000017/00000012/art01113].

3. Krogh A, Brown M, Mian IS, Sjölander K, Haussler D: **Hidden Markov models in computational biology. Applications to protein modeling.** *J Mol Biol* 1994, **235**(5):1501–1531, [http://dx.doi.org/10.1006/jmbi.1994.1104].
4. Djordjevic M, Sengupta AM, Shraiman BI: **A Biophysical Approach to Transcription Factor Binding Site Discovery**. *Genome Res.* 2003, **13**(11):2381–2390, [http://www.genome.org/cgi/content/abstract/13/11/2381].
5. Durbin R, Eddy S, Krogh A, Mitchison G: *Biological sequence analysis.* Cambridge University Press, 1st edition 1998.
6. Eddy SR: **Profile hidden Markov models.** *Bioinformatics* 1998, **14**(9):755–763.
7. Thompson JD, Higgins DG, Gibson TJ: **Improved sensitivity of profile searches through the use of sequence weights and gap excision.** *Comput Appl Biosci* 1994, **10**:19–29.
8. Gerstein M, Sonnhammer EL, Chothia C: **Volume changes in protein evolution.** *J Mol Biol* 1994, **236**(4):1067–1078.
9. Altschul SF, Carroll RJ, Lipman DJ: **Weights for data related by a tree.** *J Mol Biol* 1989, **207**(4):647–653.
10. Sibbald PR, Argos P: **Weighting aligned protein or nucleic acid sequences to correct for unequal representation.** *J Mol Biol* 1990, **216**(4):813–818.
11. Eddy SR, Mitchison G, Durbin R: **Maximum discrimination hidden Markov models of sequence consensus.** *J Comput Biol* 1995, **2**:9–23.
12. Henikoff S, Henikoff JG: **Position-based sequence weights.** *J Mol Biol* 1994, **243**(4):574–578.
13. Krogh A, Mitchison G: **Maximum entropy weighting of aligned sequences of proteins or DNA.** *Proc Int Conf Intell Syst Mol Biol* 1995, **3**:215–221.
14. Stormo G, Fields D: **Specificity, free energy and information content in protein-DNA interactions**. *Trends in Biochemical Sciences* 1 March 1998, **23**:109–113(5), [http://www.ingentaconnect.com/content/els/09680004/1998/00000023/00000003/art01187].
15. Fields D, He Yy, Al-Uzri A, Stormo G: **Quantitative Specificity of the Mnt Repressor**. *Journal of Molecular Biology* August 1997, **271**:178–194(17), [http://www.ingentaconnect.com/content/ap/mb/1997/00000271/00000002/art01171].
16. Heumann JM, Lapedes AS, Stormo GD: **Neural networks for determining protein specificity and multiple alignment of binding sites.** *Proc Int Conf Intell Syst Mol Biol* 1994, **2**:188–194.
